# Abrogation of E-Cadherin-Mediated Cellular Aggregation Allows Proliferation of Pluripotent Mouse Embryonic Stem Cells in Shake Flask Bioreactors

**DOI:** 10.1371/journal.pone.0012921

**Published:** 2010-09-23

**Authors:** Lisa Mohamet, Michelle L. Lea, Christopher M. Ward

**Affiliations:** 1 Core Technology Facility, Faculty of Medical and Human Sciences, The University of Manchester, Manchester, United Kingdom; 2 Eden Biodesign Ltd, Liverpool, United Kingdom; University of Colorado, Boulder, United States of America

## Abstract

**Background:**

A fundamental requirement for the exploitation of embryonic stem (ES) cells in regenerative medicine is the ability to reproducibly derive sufficient numbers of cells of a consistent quality in a cost-effective manner. However, undifferentiated ES cells are not ideally suited to suspension culture due to the formation of cellular aggregates, ultimately limiting scalability. Significant advances have been made in recent years in the culture of ES cells, including automated adherent culture and suspension microcarrier or embryoid body bioreactor culture. However, each of these methods exhibits specific disadvantages, such as high cost, additional downstream processes or reduced cell doubling times.

**Methodology/Principal Findings:**

Here we show that abrogation of the cell surface protein E-cadherin, using either gene knockout (Ecad-/-) or the neutralising antibody DECMA-1 (EcadAb), allows culture of mouse ES cells as a near-single cell suspension in scalable shake flask culture over prolonged periods without additional media supplements. Both Ecad-/- and EcadAb ES cells exhibited adaptation phases in suspension culture, with optimal doubling times of 7.3 h±0.9 and 15.6 h±4.7 respectively and mean-fold increase in viable cell number of 95.1±2.0 and 16±0.9-fold over 48 h. EcadAb ES cells propagated as a dispersed cell suspension for 15 d maintained expression of pluripotent markers, exhibited a normal karyotype and high viability. Subsequent differentiation of EcadAb ES cells resulted in expression of transcripts and proteins associated with the three primary germ layers.

**Conclusions/Significance:**

This is the first demonstration of the culture of pluripotent ES cells as a near-single cell suspension in a manual fed-batch shake flask bioreactor and represents a significant improvement on current ES cell culture techniques. Whilst this proof-of-principle method would be useful for the culture of human ES and iPS cells, further steps are necessary to increase cell viability of hES cells in suspension.

## Introduction

Embryonic stem (ES) cells, with their self-renewal ability and multiple lineage differentiation capacity, are attractive for many applications in regenerative medicine and drug screening. Mouse ES (mES) cells are derived from the inner cell mass of pre-implantation embryos and, although only present as a transient population *in vivo*, they can be propagated *in vitro* for extended periods when cultured in appropriate medium [1], [2]. A popular method for the culture of mouse ES cells is adherent culture in the presence of serum and the cytokine leukaemia inhibitory factor (LIF) [3], [4], although serum free media have been described [5], [6]. A fundamental element necessary to exploit the potential of ES cells in drug testing and regenerative therapies is the ability to reproducibly derive sufficient numbers of cells of a consistent quality in a cost-effective manner. Adherent methods currently employed for ES cell culture are unable to provide a suitable culture system due to the heterogeneous static conditions, resulting in batch-to-batch variation, labour intensive methodology and ultimately, restricted cell number due to the available surface area, leading to limitations in scalability [7]–[9].

Suspension bioreactors represent a cost-effective approach for the culture of cell lines and are common in industrial biotechnology applications, where nominal volumes of 25 mL to 6L are typically utilised [10]. The advantage of this culture method is the provision of a scalable, non-intensive and relatively homogenous high cell volume density microenvironment which can be easily monitored. However, undifferentiated ES cells are typically anchorage dependent and are not ideally suited to suspension culture due to the formation of cellular aggregates [9]. One method of overcoming cellular aggregation in suspension bioreactors is to utilise microcarriers to aid cell growth [7], [11]–[13]. Microcarriers exhibit a high surface-area-to-volume ratio which eliminates the surface area restriction of adherent culture techniques. However, this method also exhibits some disadvantages, including unknown effects of hydrodynamic shear stress [11], cell agglomeration (or bead bridging) as well as additional expense and down-stream purification to remove cells from the microcarrier. An alternative method is the embryoid body (EB) cultivation method, which utilises shear stress to control aggregate size [7], [14], [15] and may contain enzymatic dissociation steps to prolong culture times [16]. However, this approach is disadvantaged by diffusion limitations within individual EBs leading to EB agglomeration and less efficient cellular expansion compared to conventional culture methods.

Therefore, a suspension method that can eliminate cellular aggregation whilst providing a cost-effective approach to ES cell culture is highly desirable. We have previously demonstrated that mES cells lacking E-cadherin exhibit loss of cell-cell contact and exhibit a mesenchymal-like phenotype when grown under adherent culture conditions [17], [18]. Therefore, one mechanism for reducing cellular aggregation and EB agglomeration in mES cell suspension bioreactor culture may be the abrogation of E-cadherin protein. Fok & Zandstra (2005) [7] have demonstrated that E-cadherin is the cause of aggregation in both microcarrier and EB bioreactor culture of mES cells, however, they concluded that expression of E-cadherin protein is desirable to maintain culture robustness. Our unpublished data suggested that culture of *E-cadherin* null (*Ecad*-/-) ES cells in suspension under low shear conditions, such as in the NovaPod™ bioreactor, enabled rapid expansion of undifferentiated cells with high viability. In the current study, we show that abrogation of E-cadherin mediated cell-cell contacts in mES cells allows for robust and scalable culture of the cells in a manual fed-batch shake flasks, akin to that utilised for bacterial culture. Furthermore, EcadAb ES cells grown for 15 d in shake flask culture maintain high viability, normal karyotype and pluripotency. This is the first demonstration of a near single-cell suspension culture method allowing the expansion of undifferentiated mES cells over prolonged periods and represents a significant improvement on current ES cell culture techniques by eliminating batch-wise enzymatic passaging variation and offering significantly decreased operator times.

## Materials and Methods

### Adherent Culture of ES cells

Murine embryonic stem cell line D3 (wild type (wt) and *E-cadherin*-/- (*Ecad*-/-) ES cells [19] were maintained in an undifferentiated state as described previously unless otherwise stated [17], [20]. Cells grown in the presence of the E-cadherin (E-cad) neutralizing antibody (Monclonal Anti-Uvomorulin/E-Cadherin Clone DECMA-1; Sigma, Dorset UK) were cultured as above with supplementation of the antibody (2 µl/mL; stock concentration 6 mg/mL rat IgG1) at each passage. WtES cells exhibited a normal karyotype following both adherent (p28) and suspension culture (15 d).

### Static Suspension culture of E-cad -/- ES cells

Wt and Ecad-/- ES cells (10^6^ total cell seeding) were cultured in suspension in media lacking LIF in plastic bacteriological Petri dishes (Corning). Cell suspensions were agitated every 24 h by pipetting. Cells were passaged typically every 2 d by transfer of 2.5 mL of cell suspension into 7.5 mL of fresh medium.

### Shake flask culture of *E-cad-/-* and EcadAb ES cells

Initial experiments examined growth kinetics from variant seeding densities, agitation rates and antibody concentrations in a shake flask format for both *E-cad*-/- and EcadAb ES cells. Cultures were sampled every 24 h over a 96 h period in triplicate to determine optimal conditions for bioreactor culture. Upon optimisation, vented shake flasks (125 mL) (Corning) were inoculated with 1.0E+04 viable cells/mL (vc/mL) *E-cad*-/- ES cells in a working volume of 25 mL media and placed on a shaking platform (1″ orbit- 140 rpm; Satorius, Surrey, UK) at 37°C/5%C0_2_. Flasks were sampled every 48 h and results plotted for each sample prior to being split to 1.0E+04 vc/mL using freshly prepared media. For EcadAb ES cell culture, shake flasks were inoculated as described in a working volume of 25 mL media supplemented with 4 µl/mL DECMA-1 antibody (Sigma, Dorset, UK) under the above conditions. Flasks were sampled every 72 h and results plotted for each sample prior to cultures being split to 1.0E+04 vc/mL using freshly prepared media and antibody. Cell number and viability were determined using an automated cell counter (Innovatis) with trypan blue exclusion. Population doubling times were calculated using *Doubling Time Software v1.0.10* (http://www.doubling-time.com) (Roth, V. 2006).

### Embryoid Body Formation

Undifferentiated wtES cells were dissociated from adherent cultures with 0.05% trypsin-EDTA solution (PAA, Yeovil, UK) or directly transferred from shake flask culture at day 15. Inoculation of 1.0E+06vc/50 mL into a Novapod ™ Bioreactor (Medcell, Cambridge, UK) was used to initiate EB formation in media without LIF. Each reactor vessel was rotated at 19 rpm at 37°C/5% CO_2_. EBs formed within 48 h and maintained for 15 d by media replenishment every 2 d. On day 15, EBs were harvested for RNA isolation or plated into gelatin-treated wells and allowed to adhere overnight prior to immunofluorescent analysis.

### Human ES cell culture

Human ES cells (H1) [21] were cultured in 60 mm dishes (BD Falcon, Oxfordshire, UK) coated with 0.1%w/v gelatin and 1×10^6^ cells/dish of irradiated MF1 mouse embryonic fibroblast (MEfs) feeder cells. Cells were maintained in DMEM/F12 supplemented with 20% knockout serum replacement, 1 mM L-glutamine, 0.1 mM 2-mercaptoethanol, 1% non-essential amino acids and 4 ng/ml basic fibroblast growth factor (all Invitrogen) and incubated at 37°C/5% CO_2_. Cells were mechanically passaged every 7–9 days and transferred to new MEF feeder layers. For static suspension culture, cells were instead transferred to uncoated 60 mm dishes and media supplemented with an E-cadherin neutralising antibody (2 µg/ml SHE78.7, Invitrogen) or isotype control antibody (2 µg/ml mouse IgG2a, Invitrogen).

### Immunofluorescent imaging of ES cells

ES cells were fixed in 4% w/v Paraformaldehyde and stained in situ, as previously described [17]. Primary antibodies were as follows: mouse anti-Oct4, anti-FoxA2 (1∶100; both Santa Cruz Biotechnology, CA, USA) anti-Nanog (1∶100; Chemicon, CA), anti-E-cad (1∶100; BD Biosciences. Oxford, UK), anti-Nestin (1∶250), anti-β-III Tubulin (β-III Tub) (1∶1000) and anti-α Smooth Muscle Actin (Asma) (1∶50) (All Abcam, Cambridge, UK). The appropriate secondary antibodies conjugated with Alexa Fluors 488 or 546 were used (1∶500, Invitrogen, Paisley, UK) and all samples were mounted using DAPI Vector shield (Vector Laboratories, Peterborough, UK). The cells were viewed on a Leica DM500 fluorescence microscope.

### Fluorescent flow cytometry analysis of ES cells

Undifferentiated ES cells were dissociated from adherent culture using dissociation buffer (Invitrogen, Paisley, UK) or harvested directly from shake flask suspension cultures at day 15 (n = 3). The following method has been described previously [17]. Briefly, the cells were washed in PBS and resuspended in 0.2% w/v BSA in PBS containing the primary antibody; anti-mouse SSEA-1 (1∶100 dilution; Santa Cruz Biotechnology, CA, USA) and incubated for 30 min on ice. Cells were washed and resuspended in the appropriate phycoerythrin-conjugated secondary antibody (1∶100 dilution; Santa Cruz Biotechnology) and incubated for 30 min on ice. The cells were washed and fixed in 1% w/v paraformaldehyde. Cell fluorescence was analysed using a Becton Dickinson FACScaliber. Viable cells were gated using forward and sidescatter and all data represents cells from this population.

### RT-PCR

Total RNA was isolated from the cells using the RNeasy Kit, (Qiagen, West Sussex, UK) according to manufacturer's instructions. RNA preparations were quantified by absorbance at 260 nm (A_260_) using a Nanodrop spectrophotometer (Labtech Intl., E. Sussex, UK). Synthesis of cDNA was performed as described previously [13]. PCR was performed using 1 µl of the cDNA solution and 35 cycles at the optimal annealing temperature. Samples were run on 2% w/v agarose gels containing 400 ng/ml ethidium bromide and visualized using an Epi Chemi II Darkroom and Sensicam imager with Labworks 4 software (UVP, CA, USA). Primer sequences and annealing temperatures can be found in Soncin et al. (2009) [17].

## Results

### Static Suspension Culture of Wt and *E-cad-/-* ES Cells

**Figure 1:**
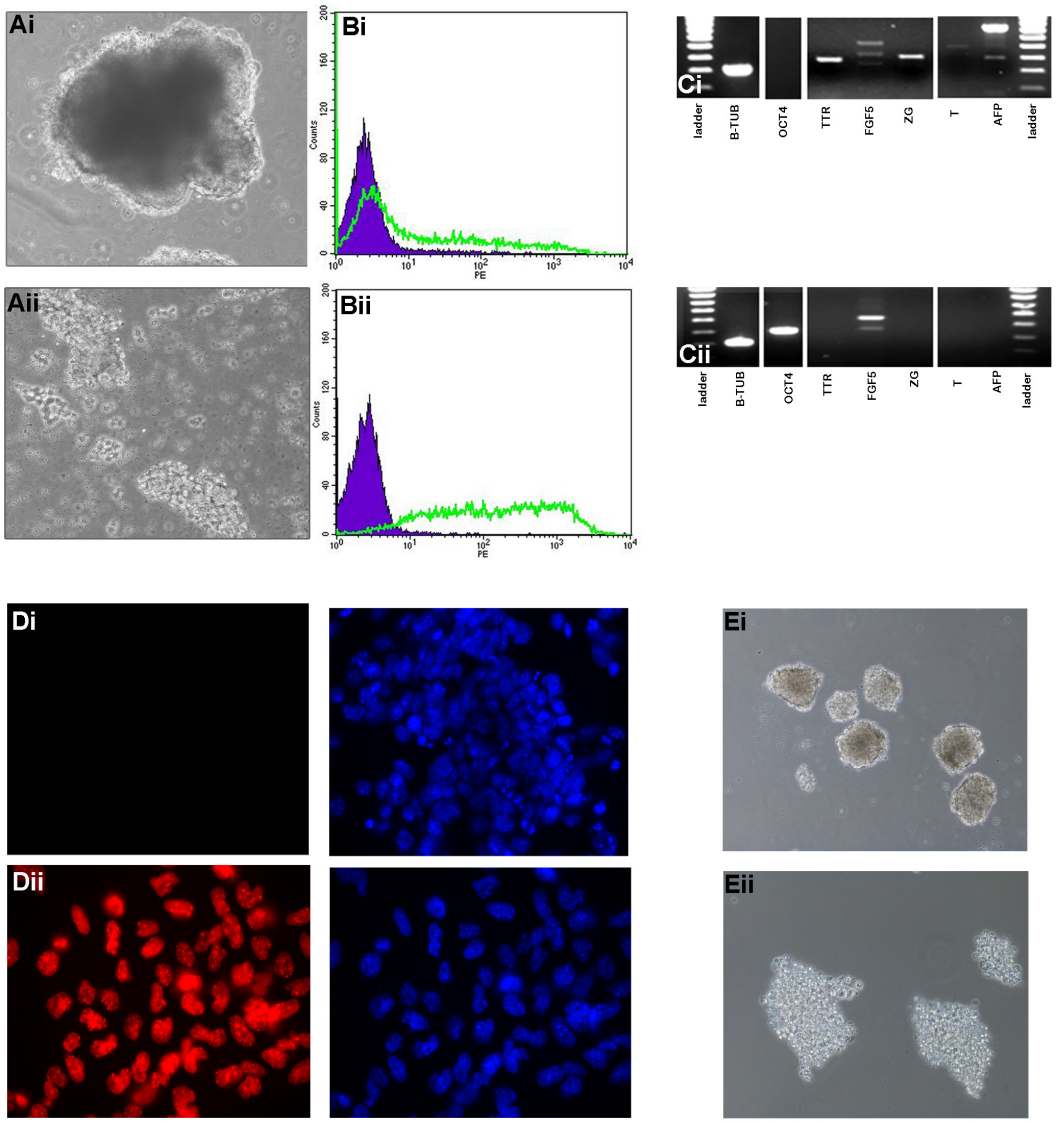
Characteristics of Ecad-/- ES cells, EB formation and Human ES cells in static suspension culture. Wt or *Ecad*-/- ES cells were cultured for 30 d in static suspension in the absence of LIF. (A) Phase contrast images showing typical EB morphology for (i) wtES cells, in contrast to the lack of adhesion in (ii) *Ecad*-/- ES cells (d5 shown for both). (B) Expression of cell surface SSEA-1 in (i) wtES cells and (ii) *Ecad*-/- ES cells cultured for 30 d without LIF was determined by FACS. SSEA-1 (green profile); isotype control antibody (purple closed profile). (C) RNA was collected from (i) wt and (ii) *Ecad*-/- ES cells cultured in suspension as above (d14 shown) and assessed for expression of *β-Tub*, *Oct-3/4*, *Ttr*, *Fgf5*, *Zg, T* and *Afp* transcripts using RT-PCR. (D) Wt and *Ecad*-/- ES cells cultured as above were plated onto gelatin-treated plates in the presence of LIF for 2 d and assessed for Nanog protein expression ((i) and (ii) respectively), total cells were visualised using DAPI. Note that *Ecad-/-* ES cells maintain expression of Nanog, whereas wtES cells do not exhibit any Nanog positive cells. (E) H1 human ES cells were cultured in static suspension for 24 h in the presence of an isotype control antibody (Ei) or an E-cadherin neutralising antibody (Eii). Cells grown in control antibody formed typical EB morphology and cell-cell contacts. However, cells grown in neutralising antibody had low viability; forming aggregates of non-viable cells.

We have previously shown that *Ecad*-/- ES cells exhibit loss of cell-cell contact and are able to self-renew in serum-supplemented medium in the absence of LIF [17]. To determine whether *Ecad*-/- ES cells would remain undifferentiated in static suspension culture in the absence of LIF, we grew the cells in bacteriological grade dishes for 30 d (Figure 1). *Ecad*-/- ES cell cultured in static suspension culture exhibited weak cell-cell interaction and failed to establish characteristic EB morphology compared to wtEBs (Figure 1A(i) and (ii), respectively). Following 30 d in suspension culture, expression of SSEA-1 was determined by fluorescent flow cytometry (Figure 1B). WtES cells exhibited low levels of SSEA-1 expression (Figure 1B(i)) whereas the *Ecad*-/- cell population exhibited >95% of the cells expressing SSEA-1 (Figure 1B(ii)). To further confirm the undifferentiated phenotype of *Ecad*-/- ES cells, we assessed expression of transcripts encoding *Oct-3/4*, and the differentiation markers *Fibroblast growth factor-5* (*Fgf5*), *Transthyretin (Ttr*), *ζ- globin (Zg*), *Brachyury (T)* and *α-fetoprotein (Afp)* following 14 d in suspension culture (Figure 1C). WtES cells exhibited expression of *Ttr*, *Fgf5*, *Zg*, *T* and *Afp* and lack of *Oct3/4* transcripts, demonstrating differentiation of a significant proportion of cells within the population (Figure 1C(i)). In contrast, *Ecad*-/- ES cells expressed *Oct3/4* transcripts and failed to upregulate any of the differentiation markers (Figure 1C(ii)). Following 30 d in suspension culture, wtES and *Ecad*-/- ES cells were cultured on gelatin-treated plates in the presence of LIF for 2 d and assessed for Nanog protein expression using immunofluorescent microscopy (Figure 1D). WtES cells showed no positive immunoreactivity for Nanog (Figure 1D(i)), whereas *Ecad*-/- ES cells maintained expression of Nanog protein in the majority of the population (Figure 1D(ii)).

### Static suspension culture of human ES cells

Since we have shown that abrogation of E-cadherin in adherent human ES cell culture results in loss of cell-cell contact without induction of differentiation [22], we assessed whether these cells could survive in static suspension culture in the presence of an E-cadherin neutralising antibody (SHE78.7) (Figure 1E). Morphological analysis following 24 h in suspension showed that control antibody-treated cells maintained cell-cell contacts and viability (Figure 1Ei); however, whilst neutralising antibody-treated cells exhibited decreased aggregation (Figure 1Eii) this was associated with very low viability.

### Effect of inoculation density on *Ecad-/-*ES cell growth and viability in shake flask culture

**Figure 2:**
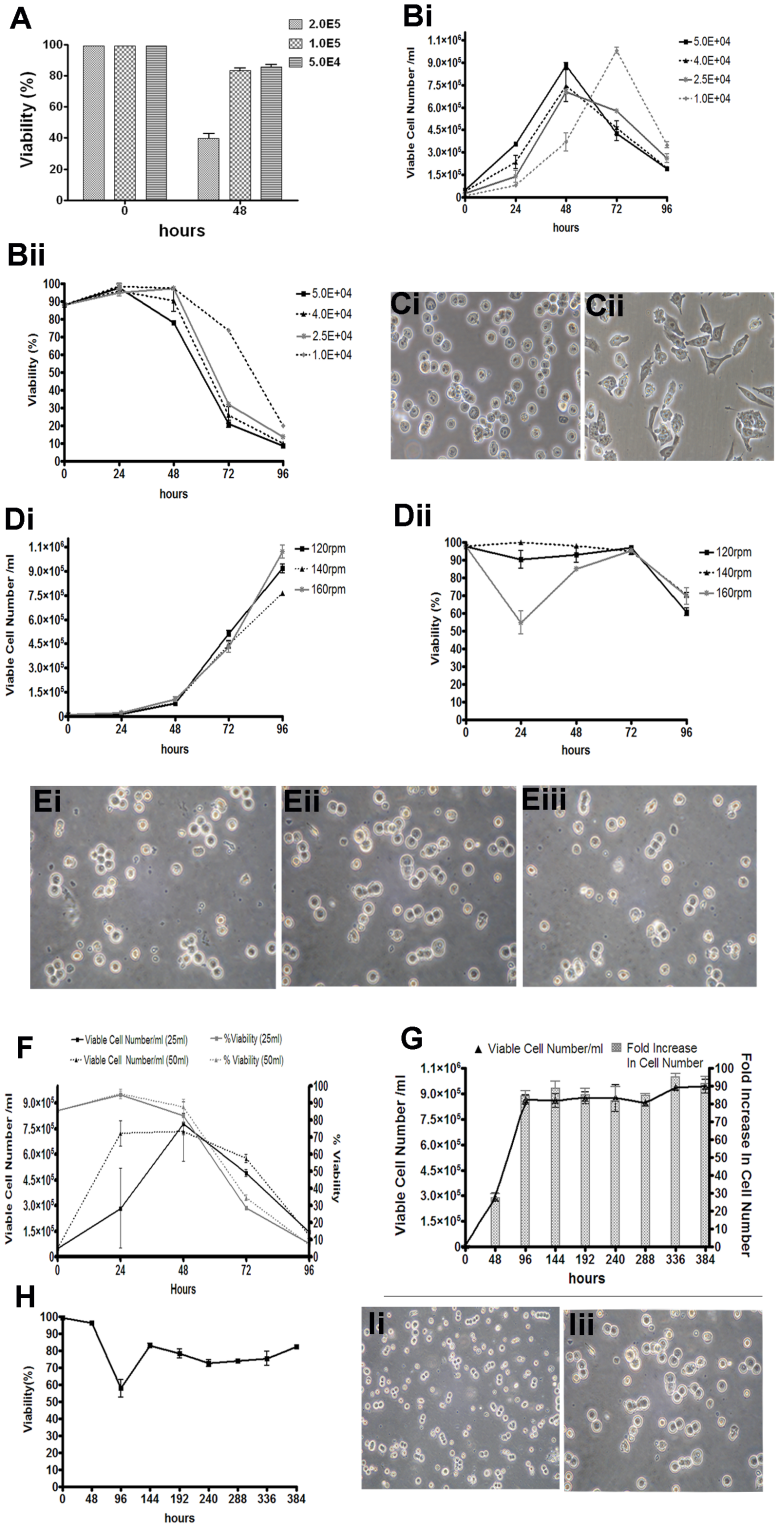
Expansion of Ecad-/- ES cells in shake flask culture. (A) *Ecad*-/- ES were seeded into shake flasks at variant densities and percentage cell viability calculated over 48 h (n = 3), demonstrating that seeding densities above 2.0E+5 vc/mL results in significant cell death at 48 h. (B) Triplicate shake flasks were inoculated with *Ecad*-/- ES cells at different densities in 25 mL of media at 140 rpm over a 96 h period. (i) Optimal expansion and cell viability was achieved using 1.0E+4 vc/mL; with a >90 fold expansion in cell numbers by 72 h and (ii) a mean cell viability of 98.5±2.1% after 48 h. (C) Phase contrast microscopy shows that cells maintained in (i) shake flask cultures have dispersed growth and are rounded in appearance (ii) in contrast to the mesenchymal phenotype of cells when grown under adherent culture conditions. (D) The effect of agitation on (i) growth kinetics of *Ecad*-/- ES cells were assessed daily for 96 h (n = 3). (ii) Agitation rates of 160 rpm were detrimental to cell viability. Fewer cell aggregates were observed at 140 rpm. (E) Phase contrast images show culture morphology of cells at (i) 160 rpm, (ii) 140 rpm & (iii) 120 rpm (48 h shown). (F) Analysis of viable numbers of *Ecad-*/-ES cells inoculated into flasks containing 25 mL or 50 mL of media (maintaining a 1∶5 working: total volume) for 96 h showed no differences in morphology or viability when scaled up. *Ecad*-/- ES cells were seeded into shake flasks at 1.0E+4 vc/mL in 25 mL media at 140 rpm and maintained for 16 d, sampled every 48 h prior to being split back to 1.0E+4 vc/mL (n = 3). (G) Analysis of viable cell numbers showed that maximal cell expansion was achieved following 96 h in culture, with a mean fold increase in cell number of 88.6±4.75 and (H) mean cell viability of 80%±2.9 for duration of the culture. (i) Phase contrast images of culture morphology were captured following 16 d in suspension (i) ×20 and (ii) ×40 magnification. Values represent mean ± SD.

Static batch culture of *Ecad*-/- ES cells was found to be an ineffective process as the loose cell aggregates required dispersing every 24 h, therefore, we investigated whether *Ecad*-/- ES cells could be cultured using a shake flask bioreactor (Figure 2). An initial agitation rate of 140 rpm was chosen as this rate is commonly used in shake flask mammalian cell culture (Lea, personal communication). Analysis of inoculation densities of 5×10^4^, 10^5^ and 2×10^5^ viable cells (vc)/mL over 48 h revealed that lower densities were more favourable for maintaining cell viability above 80% (Figure 2A). To further investigate the optimal inoculation density, we seeded shake flasks with 1×10^4^, 2.5×10^4^, 4×10^4^ and 5×10^4^ vc/mL and assessed total viable cell number (Figure 2B(i)) and percentage viability (Figure 2B(ii)) over 96 h. Peak total viable cell number was achieved at 48 h in the majority of inoculation densities, except for 1×10^4^ vc/mL, which peaked at 72 h. Mean cell viability was similar between all inoculation densities over 48 h (>90%; Figure 2b(ii)), with 1×10^4^ vc/mL exhibiting increased viability over 72 h. Overall, an inoculum of 1×10^4^ vc/mL was considered to be optimal, achieving >90 fold expansion of cell numbers over 72 h. Culture morphology showed dispersed growth of *Ecad* -/- ES cells with a rounded appearance, often observed as multiple rows of cells, indicative of actively dividing cells (Figure 2C(i); 48 h shown). In contrast, *Ecad*-/- ES cells cultured under adherent conditions exhibited a mesenchymal-like morphology, as previously described [17] (Figure 2C(ii)).

### Effect of agitation rate on *Ecad-/-* ES cell growth and viability in shake flask culture

To determine the effect of agitation on *Ecad*-/- ES cell growth and viability in shake flasks, we cultured 1×10^4^ vc/mL in 25 mL media over 96 h at 120, 140 and 160 rpm (Figure 2D). Viable cell numbers were greatest at 120 and 160 rpm following 96 h in culture (Figure 2D(i)). However, culture viability at 160 rpm decreased significantly to <60% within 24 h (Figure 2D(ii)) and, although the cell numbers continued to increase throughout the culture period, the decreased viability was considered sub-optimal. This was also reflected in culture morphology, where cellular debris and small rounded clumps of cells were observed at 160 rpm (Figure 2E(i), 48 h shown). Cell viability rates of >90% were observed at 120 rpm over 72 h and this was increased further (∼95%) at 140 rpm. Under all agitation rates, cell viability was decreased at 96 h which is likely to reflect exhaustion of essential media component(s). Agitation rates of 120 rpm and 140 rpm resulted in dispersed growth of *Ecad*-/- ES cells, with minimal aggregation (Figure 2E(ii) and (iii), respectively at 48 h). The optimal agitation rate for the culture of *Ecad*-/- ES cells was considered to be 140 rpm due to high viability of the culture over 72 h.

### Scale-up of *Ecad-/-* ES cell culture in manual batch shake flask culture

To assess the scalability of the shake flask bioreactor method, we cultured *Ecad*-/- ES cells in 50 ml working volume whilst maintaining an inoculation density of 1×10^4^ vc/mL, an agitation rate of 140 rpm and a working: total volume ratio of 1∶5 (Figure 2F). Similar cell viabilities and total viable cell numbers were obtained between 25 mL and 50 mL working volumes, demonstrating the scalability of the method.

### Culture of *Ecad-/-* ES cells in manual fed-batch shake flask culture

To determine whether a continual supply of ES cells could be achieved using a fed-batch approach over a specified time frame, shake flasks were inoculated with 1×10^4^ vc/mL in 25 ml media and maintained at 140 rpm at over a 16 d period. Flasks were sampled every 48 h, viable cell counts determined and 1×10^4^ vc/mL used as an inoculum in 25 mL of fresh medium (Figure 2G). At 48 h, total viable cell number and fold-increase in viable cells was similar to that observed in batch culture. However, at 96 h there was a significant increase in both total viable cell number and fold-increase in viable cells, suggesting the cells undergo an adjustment phase in the initial 48 h of culture. For example, at 48 h the fold increase in viable cells was 29-fold (±2.5) compared to 86.5-fold (±2.8) at 96 h. Over the subsequent culture time, viable cell number and fold increase in viable cells remained relatively constant, with a mean fold increase in cell number of 88.6±2.3 over any 48 h period. A mean cell viability of 80%±12.5 was observed over the culture period (Figure 2H), with the lowest viable cell count associated with the proposed adjustment phase at 96 h. Cell morphology throughout the culture period showed dispersed growth of *Ecad*-/- ES cells, as previously described (Figure 2I; 16 d shown at (i) ×20 and (ii) ×40 magnification). The population doubling time was calculated when viable cells exhibited exponential growth, with the resulting doubling times ranging between 7.3 h and 9.9 h. The cumulative expansion of *Ecad*-/- ES cells achieved over the 16 d culture period was 2775-fold, equating to 6.94×10^8^ total viable cells from a single 25 mL working volume.

### Culture of wtES cells (EcadAb ES cells) treated with DECMA-1 in shake flasks

**Figure 3:**
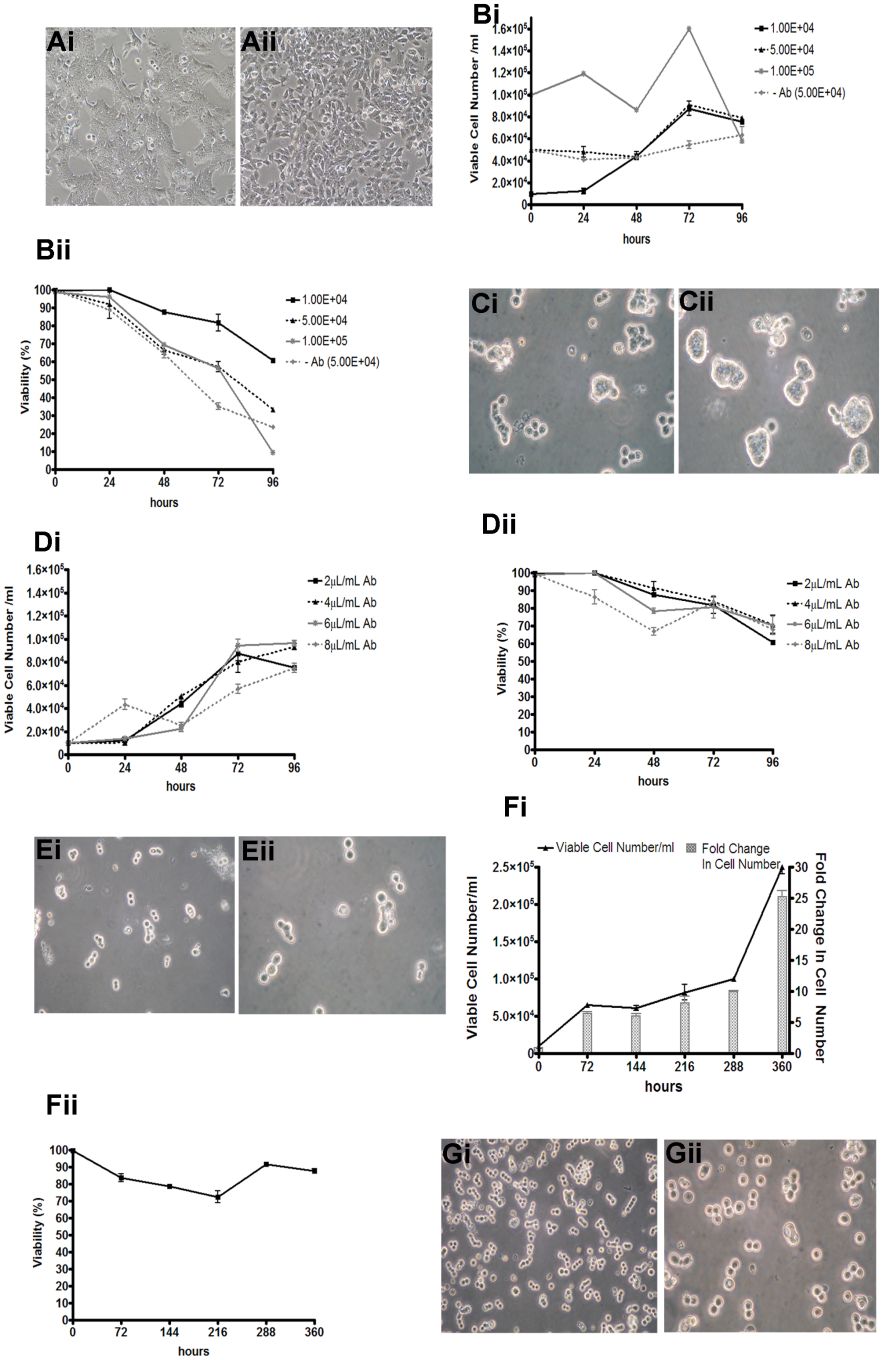
Expansion of EcadAb ES cells in shake flask culture. (A) Phase contrast images show culture morphology of (i) wtES cells grown under routine adherent culture conditions and (ii) supplemented with 2 µl/mL DECMA-1 antibody. (B) EcadAb ES cells were seeded into shake flasks at variant densities and cell counts performed daily for 96 h (n = 3). (i) Optimal expansion was achieved at 1.0E+4 vc/mL showing (ii) mean cell viability of >99%±0.1% following 24 h, decreasing to 60.6%±0.95% at 96 h. (C) Phase contrast microscopy of culture morphology shows dispersed growth in (i) EcadAb ES cells; this is in contrast to (ii) wtES cells cultured without antibody with the formation of aggregates. (D) Optimisation of the minimal antibody concentration required to maintain dispersed growth of EcadAb ES cells was examined. (i) Optimal cell expansion and (ii) viability was achieved using 4 µl/mL antibody, concentrations >6 µl/mL reduced cell viability at this cell density (n = 3). (E) Phase contrast microscopy shows that cells maintained in 4 µl/mL DECMA have dispersed growth, are rounded in appearance and can be observed in rows of cells; suggestive of actively replicating cells. (48 h shown (i) ×20 and (ii) ×40 magnification. EcadAb ES cells were seeded into shake flasks at 1.0E+4 vc/mL in 25 mL media supplemented with 4 µl/mL antibody at 140 rpm and maintained for 15 d. Cell counts were performed every 72 h prior to cultures being split back to 1.0E+4 vc/mL (n = 3). (F) (i) Viable cell numbers remained constant up to 288 h in culture, at which point viable cell numbers peaked representing a 25 mean fold expansion between 288–360 h. (ii) Mean cell viability for the duration of the culture was 86%±2.1% (G) Phase contrast images of EcadAb ES cell morphology were captured following 15 d in suspension (i) ×20 and (ii) ×40 magnification. Values represent mean ± SD.

To determine whether inhibition of E-cadherin in wtES cells would allow their culture in a shake flask bioreactor we utilised the E-cadherin neutralising antibody DECMA-1 [23], [24]. We have previously demonstrated that culture of mES cells in the presence of DECMA-1 results in loss of E-cadherin mediated cell-cell contact and decreased E-cadherin expression [18]. Prior to suspension culture, wtES cells were grown under routine adherent culture in the presence of 2 µl/mL of DECMA-1 for 4 passages to determine any cytopathological effects and allow the cells to adapt to dispersed growth. No cytopathological effects were observed in wtES cells exposed to DECMA-1 and percentage viability was >98% for each passage (Figure 3A(i) and (ii)).

EcadAb ES cells grown in adherent culture in the presence of DECMA-1 antibody as above were used to inoculate 25 mL of media with 1×10^4^ vc/mL, 5×10^4^ vc/mL or 1×10^5^ vc/mL and cultured in shake flasks at 140 rpm for 96 h. Viable cell number (Figure 3B(i)) and percentage viability (Figure 3B(ii)) were assessed every 24 h. A significant increase in cell number was detected within the 1×10^4^ vc/mL and 5×10^4^ vc/mL cultures, whereas, the 1×10^5^ vc/mL culture exhibited decreased cell numbers compared to the inoculum density. Cell viability was highest in the 1×10^4^ vc/mL cultures over 96 h, with mean cell viability of 99.0±0.1% at 24 h and 60.6±0.95% at 96 h (Figure 3B(ii)). Culture morphology throughout showed that EcadAb ES cells appeared predominantly as a single cell suspension, with rows of cells observed suggestive of actively replicating cells (Figure 3C(i); 48 h shown), although some small cell aggregates were visible within the culture. By contrast, wtES cells cultured in the absence of DECMA-1 exhibited tightly formed cell aggregates and relatively static cell numbers over the culture period (Figure 3C(ii); 48 h shown). Inoculum densities of EcadAb ES cells above 1×10^4^ vc/mL also resulted in significantly increased levels of cell aggregation (data not shown). To assess the optimal antibody concentration required to support dispersed growth and maximal viable cell densities, four concentrations of DECMA-1 antibody (2 µl-, 4 µl-, 6 µl- & 8 µl/mL) were examined over 96 h (Figure 3D) using an inoculum density of 1×10^4^ vc/mL. Total viable cell number (Figure 3D(i)) and percentage viability (Figure 3D(ii)) were assessed every 24 h. Higher concentrations of antibody (6 µl and 8 µl/mL) resulted in decreased cell viability (<80%) within 48 h (Figure 3D(ii)). This may reflect higher concentrations of sodium azide in these samples which is contained in the DECMA-1 diluent. Concentrations of 4 µl/mL of DECMA-1 resulted in high cell viability and reproducible growth kinetics of EcadAb ES cells. In addition, cultures exhibited predominantly single cell suspension growth with evidence of actively dividing cells (Figure 3E; 48 h shown (i) ×20, (ii) ×40 magnification).

### Culture of EcadAb ES cells in manual fed-batch shake flask culture

Following optimisation of the conditions for maintaining dispersed growth of EcadAb ES cells in shake flasks we assessed fed-batch culture of the cells over 15 d (Figure 3F). Triplicate flasks were inoculated with 1×10^4^ vc/mL in 25 mL media supplemented with 4 µl/mL DECMA-1 antibody and maintained at 140 rpm. Flasks were sampled every 72 h and viable cell number (Figure 3f(i)) and percentage viability (Figure 3F(ii)) determined. Every 72 h, a proportion of the population was utilised to provide a fresh inoculum of 1×10^4^ vc/mL. Viable cell number and fold-change in cell number demonstrates two distinct stages over the 15 d culture period, similar, albeit delayed, to that observed with Ecad-/- ES cells (Figure 2G). During the first 288 h of culture the cells exhibited an average of 7.7±1.8-fold increase in cell numbers and relatively similar total viable cell numbers every 72 h (Figure 3F(i)). This is consistent with that observed for wtES cells cultured in adherent culture in the presence of 4 µl/mL of DECMA-1 (data not shown). After 360 h, total viable cell number and fold-change in cell number was increased 2.5-fold, equating to a mean fold increase in cell number of 25.3±0.9 over a 72 h period. Mean cell viability was 86%±2.1% (Figure 3F(ii)) for the duration of the culture period. Culture morphology was similar to that observed for *Ecad*-/- ES cells; dispersed growth with a rounded appearance, often observed in rows, indicative of actively dividing cells (Figure 3G; 15 d, (i) ×20 and (ii) ×40 magnification). Population doubling times were calculated as previously, resulting in doubling times ranging between 15.6 h–27.1 h. Karyotype analysis confirmed that following 15 d in suspension culture no abnormalities were present in EcadAb ES cells. The cumulative expansion of EcadAb ES cells achieved was 133-fold over the 15 d culture period, equating to 1.33×10^6^ total viable cells from a single 25 mL working volume. However, using cells adapted to suspension culture, the total viable cell count over 15 d would reach approximately 3×10^8^ cells from a single 25 mL working volume. Under adherent cell culture conditions, forty T75 cell culture flasks would be necessary to culture an equivalent number of cells.

### EcadAb ES cells maintain an undifferentiated phenotype in shake flasks

**Figure 4:**
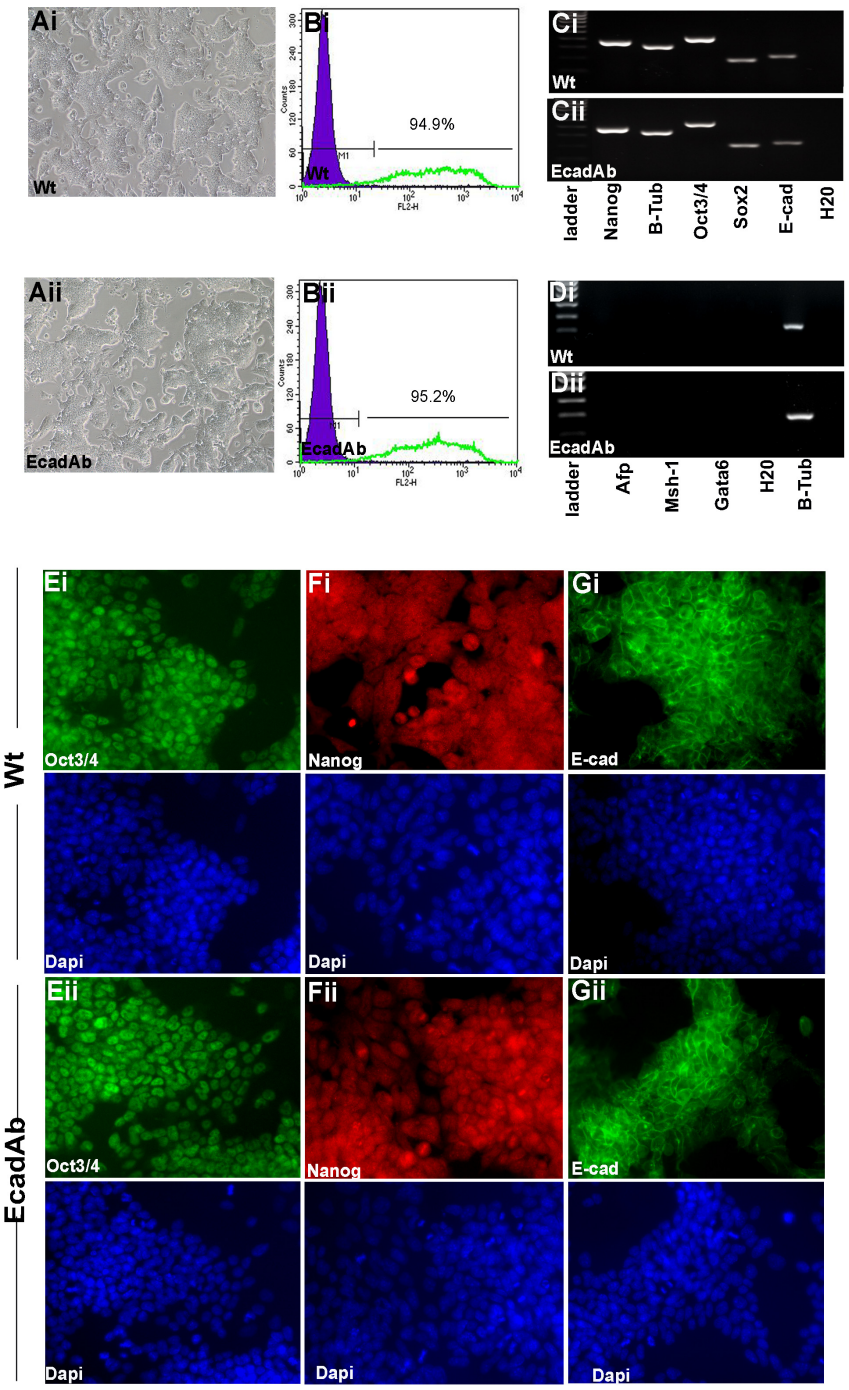
EcadAb ES cells grown in shake flasks maintain an undifferentiated phenotype. EcadAb ES cells grown in shake flasks for 15 d or wtES cells grown under standard adherent conditions at an equivalent passage number were transferred to gelatin-treated plates and allowed to adhere overnight prior to analysis. (A) Phase contrast images show that both (i) wt and (ii) EcadAb ES cells exhibit typical ES cellular morphology. (B) Assessment of SSEA-1 expression by FACS analysis (n = 3) shows that in (i) wt and (ii) EcadAb ES cells, ∼95% of cells are SSEA-1 positive (green profile). Isotype control antibody (purple closed population). (C) RT-PCR analysis was performed on (i) wt and (ii) EcadAb ES cells to determine expression of pluripotency markers and (D) differentiation markers. Immunofluorescent analysis of (E) Oct3/4, (F) Nanog, (G) E-cad and DAPI in (i) wtES cells and (ii) in EcadAb ES cells. All images captured at ×20 magnification.

To determine if EcadAb ES cells grown in manual fed-batch culture over 15 d maintain an undifferentiated phenotype we examined expression of a number of markers of pluripotency. EcadAb ES cells grown in shake flasks for 15 d were plated onto gelatin-coated plates and allowed to adhere under routine culture conditions in the absence of DECMA-1 antibody prior to analyses. EcadAb ES cells exhibited similar colony morphology to cells grown in adherent culture (designated wtES cells) (Figure 4A(i) and (ii); d4 shown). Furthermore, wt and EcadAb cells exhibited similar expression levels of cell surface SSEA-1, as determined by fluorescent flow cytometry analysis (Figure 4B(i) and (ii) respectively). The undifferentiated phenotype of wt and EcadAb ES cells was further verified by transcript expression analysis of *Oct3/4*, *Nanog*, *E-cadherin* and *Sox-2* ((Figure 4C(i) and (ii) respectively). Furthermore, both wt and EcadAb ES cells lacked expression of the differentiated cell lineage markers; *Afp*, *Musashi-1* (*Ms1*) and *GATA binding protein 6* (*Gata6*) (each representative of one of the three germ layers; Figure 4D(i) and (ii) respectively). The undifferentiated phenotype of wt and EcadAb ES cells was also validated at the protein level with positive immunoreactivity for OCT3/4 (Figure 4E(i) and (ii) respectively), NANOG (Figure 4F(i) and (ii) respectively) and E-CADHERIN (Figure 4G(i) and (ii) respectively). Total cells were visualised by DAPI with corresponding images shown.

### Differentiated EcadAb ES cells express markers of the three primary germ layers

**Figure 5:**
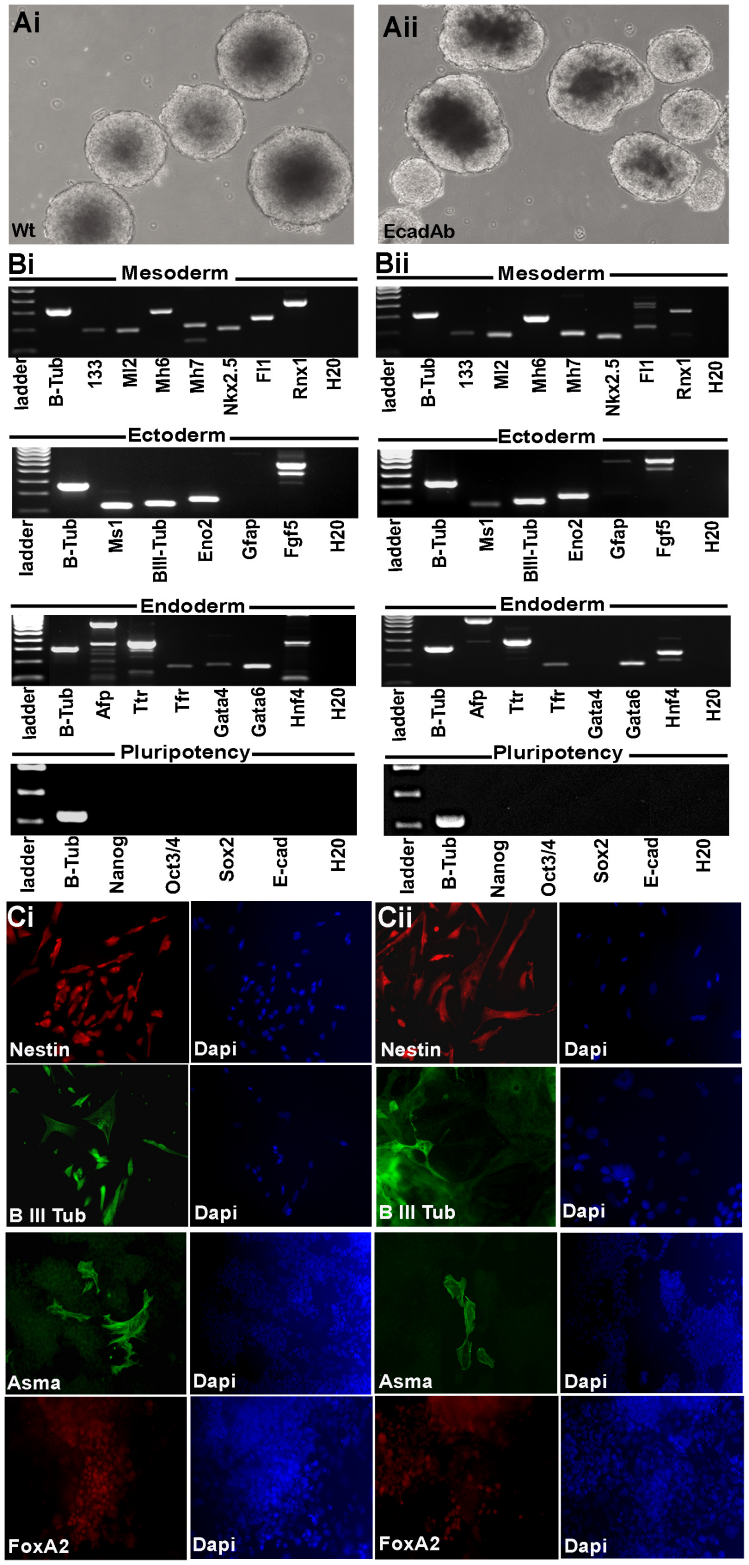
EcadAb ES cells grown in shake flask culture maintain their differentiative capacity in vitro. To determine whether EcadAb ES cells retain a normal differentiative capacity *in vitro* upon withdrawal of antibody, wtES and EcadAb cells were cultured in media without LIF for 15 d in a Novapod ™ bioreactor. (A) Phase contrast images showing formation of (i) wt derived EBs and (ii) EcadAb derived EBs (d3 shown). (B) RT-PCR analysis was performed on d15 EBs derived from (i) wt ES cells and (ii) EcadAb ES cells for differentiation lineage markers labelled mesoderm, ectoderm, and endoderm. (i) Wt and (ii) EcadAb ES cells did not express *Nanog*, *Oct-3/4*, *Sox2* and *E-cad* transcripts (labelled pluripotency). (C) Immunofluorescent analysis of markers representative of ectoderm (Nestin and β-III Tub), mesoderm (Asma) and endoderm (FoxA2) in both (i) wt and (ii) EcadAb ES cells. Total cells were visualised with DAPI. All images are shown at ×20 magnification.

To determine whether EcadAb ES cells maintain a pluripotent phenotype when cultured in a manual fed-batch shake flask over 15 d, wt and EcadAb ES cells were cultured in media without LIF for 15 d in a Novapod ™ bioreactor to stimulate formation of EBs. EBs formed within 48 h of culture in the Novapod™ bioreactor, with EcadAb ES cells forming EBs at a comparable rate with no obvious differences in morphology or size compared to wtES cell derived EBs (Figure 5A(i) and (ii) respectively; d3 shown). The ability of these EBs to differentiate into cells representative of the three germ layers was assessed by transcript expression analysis of differentiated cell lineage markers (Figure 5B(i) and (ii); labelled mesoderm, ectoderm and endoderm). The majority of the markers were expressed by both wt and EcadAb EBs with the exception of *Gfap*, which was absent in the former, and *Gata4*, which was absent in the latter. Furthermore, both wt and EcadAb ES cells did not express pluripotency markers as shown by RT-PCR analysis (Figure 5B(i) and (ii); labelled pluripotency). For simplicity, we have included *E-cadherin* and *Sox2* transcripts within the ‘pluripotency markers’ section of Figure 5B, although the former is expressed in differentiated epithelium and the latter can be expressed in ectodermal lineages [23], [25]. The differentiated phenotype of wt and EcadAb ES cells was also validated at the protein level with positive immunoreactivity for NESTIN, β-III TUB, α-SMA and FOXA2 proteins (Figure 5C(i) and (ii) respectively). Overall, these results demonstrate that EcadAb ES cells cultured in a manual fed-batch shake flask over 15 d retain the ability to differentiate into cells representative of the three primary germ layers.

## Discussion

A fundamental element necessary to exploit the potential of ES cells in drug discovery applications and regenerative medicine is the translation of cost-effective cell culture protocols into large scale cell expansion methods. Although major advances in bioreactor culture of mouse and human (hES) cells have been made in recent years, there remain significant disadvantages with current methods, particularly in relation to the effect of cellular aggregation on scalability. ES cells readily form aggregates when placed into suspension culture and the formation of these EBs in the absence of LIF is an effective method for induction of differentiation of mES cells [26]. Although numerous studies have demonstrated that bioreactor culture of mES cells as aggregates retain pluripotency when cultured under specific conditions, the efficiency of these protocols are restricted by potential cellular necrosis within the centre of the aggregate and, more importantly, unwanted spontaneous differentiation of the cells [27]. Furthermore, enzymatic dissociation required for passaging of these aggregates results in significant cell loss/death. Encapsulation methods are disadvantaged by increased costs in down-stream processes needed to separate the cells from the microcarrier. Our proof-of-principle experiments demonstrate a novel technology for the expansion of pluripotent mES cells as a near-single cell suspension within a manual fed-batch single closed system.

In the current study, we initially assessed the phenotype of *Ecad*-/- ES when grown under static suspension culture conditions. *Ecad*-/- ES cells failed to form characteristic EB morphology compared to wtES cells following 30 d in culture. This result supports the observation that *Ecad-*/- ES cells exhibit loose cell-cell contact [19] and that E-cadherin is responsible for cellular aggregation in suspension [7], [13]. Culture of *Ecad*-/- ES cells in shake flasks resulted in proliferation and maintenance of an undifferentiated cell phenotype. The doubling times observed for *Ecad*-/- ES cells were less than half of that reported for wtES cells grown in various suspension culture formats [7], [13], [14], [16], [28]. These results demonstrate that abrogation of E-cadherin mediated cell-cell contact results in increased ES cell proliferation. This corroborates our previous observations in adherent culture, where *Ecad*-/- ES cells exhibited a 1.7-fold increase in proliferation compared to wtES cells (Ward, unpublished data).

Several studies have suggested that inhibition of E-cadherin in suspension culture of ES cells would be detrimental to cell viability. Fok and Zandstra (2005) [7] concluded that expression of E-cadherin protein is desirable to maintain ES cell culture robustness. They reported that *Ecad*-/- ES cells were unable to maintain viability when propagated at 100 rpm in spinner flask cultures. This discrepancy may be due to increased shear forces acting upon the cells in spinner flask cultures compared to shake flasks. Dang et al (2004) [13], showed significant inhibition of EB agglomeration following treatment of established EBs with DECMA-1 in suspension culture. Therefore, E-cadherin is involved in both cell-cell and EB-EB contact, demonstrating the advantage of abrogation of this protein at the outset of suspension culture. However, Dang et al (2004) [13] suggested that the use of E-cadherin blocking antibodies in culture may adversely affect cell differentiation due to the critical function of E-cadherin in tissue organisation and regulation of gene expression. We have already demonstrated that abrogation of E-cadherin in mES cells does not affect their pluripotency [17], [18]. It has been suggested that mES cells are anchorage dependent and require appropriate support for suspension culture [11]. Our data demonstrates that this cannot be true since culture of EcadAb ES cells in suspension does not adversely affect either viability or proliferation of the cells. Indeed, given the shorter doubling times of our EcadAb ES cells when adapted to suspension culture, it is more likely that E-cadherin-mediated cell-cell contact negatively regulates ES cell proliferation in adherence culture.

Culture morphology of EcadAb ES cells in shake flasks showed that the cells appeared predominantly as a near-single cell suspension with minimal cell aggregation. A pronounced peak in total viable cell number was observed following 13 d in culture, indicating that the cells undergo an adjustment phase. However, this adjustment phase was not unique to EcadAb ES cells since *Ecad*-/- ES cells also exhibited a significant peak in total viable cell number at 96 h. Adjustment phases in mammalian cell culture are common and can be induced by changes in growth medium [5] and substratum [29]. Maximum EcadAb ES cell expansion was achieved following 14 d in culture, with a mean fold-increase in cell number of 25.3±0.9 over a 72 h period with resultant population doubling times ranging between 15.6 h and 27.1 h. These doubling times are shorter than those reported for wtES cells grown as suspension aggregates. For example, Fok et al (2005) [7] reported doubling times of 24-39 h for wtES cells grown as aggregates in spinner flask culture and, more recently, Kehoe et al (2008) [30] demonstrated the expansion of mES cells in serum free media achieved a 20-fold increase in cell number over 96 h. However, one group report expansion of pluripotent wtES cells as aggregates in stirred bioreactor cultures with doubling times of ∼16 h, yet only yielding an approximate 31-fold increase in cell numbers over 5 d [14]. In a similar study, zur Neiden et al (2007) [16] reported doubling times of 15 h during early passaging of their ES cell aggregates. However, these aggregates required trypsinisation every 4 d. Therefore, near-single cell suspension culture of mES cells in shake flasks is as efficient as the best methods described, but offers the advantage of not been subjected to batch-wise enzymatic passaging variation and is a significant improvement compared to many current methods of ES cell culture.

A suspension method that can eliminate cellular aggregation whilst providing a cost-effective approach for hES or iPS cell culture is highly desirable and the technology presented in this study should be relevant to the suspension culture of both. We have previously shown that inhibition of E-cadherin in hES cells using a neutralising antibody under adherent culture conditions results in loss of cell-cell contacts without affecting cellular viability or inducing differentiation of the cells [22]. Therefore, the ability to culture hES or iPS cells as near-single cell suspensions in shake flask bioreactors would revolutionise the stem cell field by providing a user friendly, adaptable and scalable system. However, some issues remain to be overcome, such as replacement of the antibody-mediated E-cadherin antagonist. Dang et al (2004) [13] stated that the use of blocking antibodies is not a cost-effective method for bioreactor culture of mES cells and this is true in the system described here. We are currently utilising peptide inhibitors of E-cadherin to provide cross-species reagents for the shake flask culture of ES cells. A difficulty we have observed with hES cells is their low viability in suspension culture. For example, H1 cells cultured in suspension in the presence of an E-cadherin neutralising antibody resulted in significant cell death after 24 h. Therefore, the culture of hES cells as a near-single cell suspension is likely to require additional factors to aid cell survival. One such factor is the small molecule ROCK inhibitor Y27632, which has been demonstrated to increase viability of dissociated hES cells [31], [32]. Whilst Y27632 can prolong hES cell survival in suspension culture, our intial data suggests that additional factors will be necessary to allow the derivation of large numbers of viable and undifferentiated hES cells over an extended period of time.
